# CD8+T Cell-Related Gene Biomarkers in Macular Edema of Diabetic Retinopathy

**DOI:** 10.3389/fendo.2022.907396

**Published:** 2022-07-22

**Authors:** Jing Huang, Qiong Zhou

**Affiliations:** Department of Ophthalmology, The First Affiliated Hospital of Nanchang University, Jiangxi Center of National Ocular Disease Clinical Research Center, Nanchang, China

**Keywords:** diabetic retinopathy, diabetic macular edema, CD8+T cell, bioinformatic analysis, biomarker

## Abstract

**Background:**

CD8+T lymphocytes have a strong pro-inflammatory effect in all parts of the tissue, and some studies have demonstrated that its concentration in the vitreous increased significantly, suggesting that CD8+T cells play a pivotal role in the inflammatory response of diabetic retinopathy (DR). However, the infiltration of CD8+T cells in the DR retina, especially in diabetic macular edema (DME), and its related genes are still unclear.

**Methods:**

Download the GSE16036 dataset from the Gene Expression Omnibus (GEO) database. The ImmuCellAI program was performed to evaluate the abundance of 24 immune cells including CD8+T cells. The CD8+T cell-related genes (DECD8+TRGs) between non-proliferative diabetic retinopathy (NPDR) and DME were detected *via* difference analysis and correlation analysis. Enrichment analysis and protein-protein interaction (PPI) network mapping were implemented to explore the potential function of DECD8+TRGs. Lasso regression, support vector machine recursive feature elimination (SVM-RFE), CytoHubba plug-in and MCODE plug-in in Cytoscape software, and Weighted Gene Co-Expression Network Analysis (WGCNA) were performed to comprehensively analyze and obtain Hub DECD8+TRGs. Hub DECD8+TRGs expression patterns were further validated in other two DR-related independent datasets. The CD8+TRG score was defined as the genetic characterization of Hub DECD8+TRGs using the GSVA sample scoring method, which can be administered to distinguish early and advanced diabetic nephropathy (DN) as well as normal and DN. Finally, the transcription level of DECD8+TRGs in DR model mouse were verified by quantitative real-time PCR (qPCR).

**Results:**

A total of 371 DECD8+TRGs were identified, of which 294 genes were positively correlated and only 77 genes were negatively correlated. Eight genes (IKZF1, PTPRC, ITGB2, ITGAX, TLR7, LYN, CD74, SPI1) were recognized as Hub DECD8+TRGs. DR and DN, which have strong clinical correlation, have been proved to be associated with CD8+T cell-related hub genes by multiple independent data sets. Hub DECD8+TRGs can not only distinguish PDR from normal and DN from normal, but also play a role in the early and progressive stages of the two diseases (NPDR vs DME, Early DN vs Advanced DN). The qPCR transcription level and trend of Hub DECD8+TRGs in DR mouse model was basically the same as that in human transcriptome.

**Conclusion:**

This study not only increases our understanding of the molecular mechanism of CD8+T cells in the progression of DME, but also expands people’s cognitive vision of the molecular mechanism of crosstalk of CD8+T cells in the eyes and kidneys of patients with diabetes.

## Introduction

Diabetes mellitus (DM) is a common metabolic disorder syndrome involving multiple organs in the body (1). Diabetic retinopathy (DR), one of the most common complications of DM, is a neurovascular disease characterized by destruction of the retinal neurovascular unit (NVU) caused by hyperglycemia (2). DM causes retinal microvascular leakage and occlusion, as well as a series of secondary fundus diseases, among which diabetic macular edema (DME) is the main cause of moderate and severe vision loss (3, 4). Since the 21st century, the estimated prevalence of diabetes among adults has more than tripled over two decades, from an initial estimate of 151 million (4.6% of the global population at the time) to 537 million (10.5%) today. Without adequate effective response interventions, 643 million people are projected to have diabetes by 2030 (11.3% of the population), and that number will jump to a frightening 783 million people (12.2%) by 2045 (5). According to the systematic review, there will be 160.5 million people with DR, 44.8 million people with vision-threatening diabetic retinopathy (VTDR) and 28.61 million people with DME in 2045 (6). A study shows that only 44.7 percent of Americans with DME know that diabetes has affected their visual function, while nearly 60 percent of them lack awareness of the risk of vision loss caused by diabetes ocular complications (7). DR and related complications, including DME, have brought heavy economic burden to all countries (8). Although intravitreal anti-VEGF therapy has replaced laser therapy as the first choice for significantly improving DR, there are still a large proportion of patients, especially patients with vision-threatening DME, who do not respond to this therapy (9, 10). Therefore, it is urgent and necessary to dig up new biomarkers related to the occurrence and progression of DME.

With the advancement of DR research, people realize that inflammation is indispensable in the formation of DR, and the inflammatory changes occur before the development of microvascular lesions characterized by neovascularization (11, 12). The main mechanisms of inflammatory reaction in DR are leukocyte stasis, infiltration of innate immune cells (such as macrophages and neutrophils) and adaptive immune cells (such as B and T cells), activation of microglia, complement coagulation cascade, upregulation of cytokines and increment of chemokine composition (13–15). It is well known that inflammatory mediators up-regulate first in the retina and then diffuse into the vitreous (16, 17). The vitreous body is composed mainly of water and gelatin, which consists mainly of collagen microfiber scaffolds and interwoven macromolecules of hyaluronic acid, with only a few hyalocytes in between (18). The vitreous body of normal people is in a state of immune privilege, so there is generally no leucocytes within (19). However, when the blood-retinal barrier (BRB) is disrupted, as in the pathogenesis of DR, leukocytes will enter the vitreous *via* the damaged barrier (20). T lymphocytes were observed in vitreous samples from most patients with proliferative diabetic retinopathy (PDR), but not in vitreous samples from non-diabetic patients (21). T lymphocytes were also discovered in fibrovascular membrane samples of patients with PDR, which was positively correlated with the grade of retinopathy and visual prognosis (22, 23). Vitrectomy enables ophthalmologists to easily obtain vitreous samples from DR patients, but not retinal samples, which is why there are many studies on vitreous T cells, but rare studies on retinal T cells (24). It has been demonstrated that CD8+T cells are elevated in all stages of DR and in vitreous with or without vitreous hemorrhage (21, 25). It is reasonable to conclude that CD8+T cells, as an important member of adaptive immune T cells, are involved in the formation mechanism of DR and DME, and their infiltration status in the retina deserves further investigation.

With the popularization of the next generation sequencing technology, the transcriptome research method of ribonucleic acid sequencing (RNA-seq) makes it easier to obtain gene diagnostic markers and therapeutic targets of DR and DME. At present, several studies on biomarkers of DR have been reported, but there are few reports about DME (26, 27). However, the related genetic markers of CD8+T cells, especially the direct research evidence obtained from the retina, and the molecular mechanism of CD8+T cells involved in DR and DME are not clear. In order to study the above research topics, the ImmucellAI algorithm was applied to estimate the constituent of 24 kinds of immune cells, including CD8+T cells. R software was utilized throughout the study: Firstly, the CD8+T-cell related gene (DECD8+TRGs) present between non-proliferative diabetic retinopathy (NPDR) and DME was mined *via* difference and correlation analyses. Secondly, conventional enrichment analysis was performed and a protein-protein interaction network (PPI) was plotted to explore the potential functions and pathways of DECD8+TRGs, and four different methods were introduced to comprehensively analyze and obtain Hub DECD8+TRGs. Thirdly, Hub DECD8+TRGs were verified on DR and DN-related independent datasets in two different ways. Finally, Hub DECD8+TRGs was applied to animals for *in vivo* experimental verification. *Via* the above research program, we targeted the hub genes highly related to CD8+T cells, which contributed to the genetic and molecular mechanism of the occurrence and development of DR and DME. Meanwhile, we also preliminarily discussed the crosstalk relationship between the occurrence and development of diabetes mellitus in DME and DN, and provided a new research idea for the study of the infiltration mechanism of CD8+T cells in the eyes and kidneys of patients with diabetes.

## Materials and Methods

### GEO Dataset Processing

Log in to the Gene Expression Omnibus (GEO) database (https://www.ncbi.nlm.nih.gov/geo/) and download the GSE160306 dataset. This high-throughput sequencing expression profile analysis data set is based on the GPL20301 Illumina HiSeq 4000 (Homo sapiens) platform and contains 79 DR-related transcriptome samples data covering all major stages. In this study, the gene expression profile data of NPDR (9 macular samples) and DME (10 macular samples) from two different clinical stages were assigned for analysis, and the data were normalized by Log2 transformation before analysis.

### NPDR and DME Macular Samples Immunocytes Infiltration

The abundance of 24 kinds of immune cells, including CD8+T cells, was estimated from RNA-seq digital gene expression matrix data by the Immunne Cell Abundance Identifier (ImmuCellAI), and the corresponding immune cell infiltration matrix was obtained. Download the calculated landscape data of macular immune cell infiltration in patients with NPDR and DME. The proportion of each immune cell subtype was extracted from the sample data. Heat maps of 24 types of immune cells including CD8+T cells were created using R package Pheatmap. Twenty-four types of immunocytes were divided into two layers: the first layer contained 10 immunocytes and the second layer contained 14 immunocytes. The total infiltration score of each sample in each layer was defined as 1, which is the sum of the percentages of infiltrated immunocytes in each layer. Wilcoxon rank sum test was used to compare the abundance of various immune cell types in NPDR and DME samples. The infiltration scores of CD8+T cells in the two groups were compared by Welch paired t-test.

### Identification of DECD8+TRGs

Differential expression genes (DEGs) between NPDR and DME samples were identified and obtained by edgeR package in R software. The significant DEGs standard was defined as: | logFC | (fold change) > 1.0 or p < 0.05. Pearson correlation analysis of statistically significant DEGs expression and CD8+T cell abundance was performed to determine DECD8+TRGs. The Pearson correlation coefficient (PCC) of DEGs >0.6 were recognized as DECD8+TRGs.

### Functional Enrichment Analysis

ShinyGO (http://bioinformatics.sdstate.edu/go/) is an integrated, web-accessible analytical program that graphically visualizes genetic enrichment analysis results and gene characteristics, providing an informatics reference for users to explore the biological significance of a gene set if it exists. In order to clarify the biological function and significance of DECD8+TRGs in DR and DME, the biological process of gene ontology (GO), Kyoto Encyclopedia of Gene and Genome (KEGG) and Reactome pathway were all included in the ShinyGO program for enrichment analysis. The significance threshold of enrichment analysis was p < 0.05.

### Screening Hub DECD8+TRGs by Comprehensive Methods

In this study, four methods were utilized to screen Hub DECD8+TRGs: Cytoscape software (including applications of CytoHubba plug-in and MCODE plug-in), Lasso regression analysis, support vector machine recursive feature elimination (SVM-RFE) and weighted gene co-expression network analysis (WGCNA). The functions of the two plug-ins in Cytoscape software are as follows: CytoHubba is a plug-in that contains a variety of algorithms to estimate network characteristics and rank nodes in a specified network, which could be performed to filter and identify the central elements of a complex network; the MCODE plug-in has the ability to locate one or more clusters (highly interconnected areas) in a complex network. In order to identify potential Hub DECD8+TRGs, 11 algorithms including BottleNeck, Stress, Betweenness, Radiality, Closeness, EcCentricity, EPC, Degree, MNC, DMNC, MCC in CytoHubba plug-in were implemented to record the intersection of the top 50 nodes of each method. Among the 11 algorithm conditions, only the DECD8+TRGs that meets 9 or more conditions will be recorded and recognized as candidate Hub CytoHubba genes. LASSO regression is utilized to minimize extra redundancy and eliminate irrelevant elements to achieve complexity adjustment and variable screening. R package glmnet was invocated for Lasso-Cox regression modeling analysis. The response type was deployed to binomial, the penalty coefficient was set and adjusted to 3-fold cross-validation, and lambda (λ) with minimal classification error was uncovered and manipulated to determine the variables. SVM-RFE is a feature optimization selection algorithm, which is based on support vector machine and sorts gene features according to the recursive feature deletion sequence. It tries to extract the key information genes in the classification issue and achieves high performance at the same time. R package e1071 was applied to process SVM-RFE classifier, which could classify and analyze candidate gene biomarkers. SVM-RFE model based on radial basis function and 10-fold cross validation was established, and the optimal variables were screened according to the minimum error value of 10×CV. The genes obtained by CytoHubba, SVM-RFE and Lasso were overlapped by pairwise, and the results were summarized and named as candidate hub genes.

All DECD8+TRGs between the NPDR group and the DME group were analyzed by the limma package. All genes were ranked according to the significance of differential expression and were included as candidate genes for analysis. The co-expression network was constructed by the WGCNA package. Cluster analysis was actualized by performing hclust function, and if there are outlier samples in the data set, they would be eliminated. The appropriate soft threshold (β) was determined by pickSoftThreshold function, and the approximate scale-free network distribution with fitting index R^2^ > 0.9 was obtained. The one-step network construction and module detection were accomplished by using blockwiseModules function to generate co-expression gene module and Topology Overlap Matrix (TOM) with minimum module size of 30 and cutting height of 0.25. Principal component analysis was performed on each module, and the eigenvalue (module eigengenes, MEs) of the gene module was calculated by the first principal component. The correlation coefficient between MEs and clinical features of the disease was calculated, and the gene modules which could distinguish the significant correlation between NPDR and DME were identified. The candidate Hub genes mentioned above were intersected with all the significant clinical feature module genes of WGCNA, then the obtained genes were analyzed by MCODE plug-in of Cytoscape software, and the resulting genes were recognized as Hub DECD8+TRGs.

### PPI Network Construction

STRING (search tool for searching interacting genes/proteins) is an online tool for visualizing protein-protein interactions (PPI) networks, which can be operated to search for known and predict protein-to-protein interactions. It has the ability to provide systematic screening of interactions between human proteins and genes. Upload the DECD8+TRGs list that has been analyzed for differences and correlations to the PPI tab of the STRING database to identify and construct a PPI network with the default comprehensive score of more than 0.4. Use the Cytoscape 3.9.0 software to read the PPI network results list file exported from the STRING database and re-visualize it. In the process of graphic visualization, the visual attributes of correlation coefficient and fold change were added to each displayed DECD8+TRGs.

### Validation of Hub DECD8+TRGs in DR

In order to study the relationship between Hub DECD8+TRGs and DR progress, the Pheatmap package in R was handled for unsupervised hierarchical cluster analysis of Hub DECD8+TRGs in GSE160306 dataset. Two independent datasets containing samples extracted from DR neovascularization membrane, GSE94019 (n=13) and GSE60436 (n=6), were downloaded to verify the expression of Hub DECD8+TRGs in the extramacular proliferative membranes. In order to establish a validation set comparison standard for GSE94019 and GSE60436, we still used dataset GSE160306 for analysis, but the control group was changed from NPDR to normal retina without diabetes, and the DME group remained unchanged. The split violin plot presented the comparison of expression levels between the two groups.

### Using GSVA Method to Verify the Diagnostic Characteristics of Hub DECD8+TRGs in DN

Gene set variation analysis (GSVA) is a non-parametric and unsupervised analysis method, which is mainly operated to evaluate the gene set enrichment results of microarray and RNA-seq transcriptome. As an analytical method, it is usually used to study the differences of some gene sets of interest among different samples, or to seek for some important gene sets, so as to illustrate the causes of phenotypic differences from the perspective of bioinformatics. In this study, Hub DECD8+TRGs was adopted as a feature gene set to participate in the verification of each DN dataset, and R package GSVA was applied to evaluate the GSVA enrichment score of each independent sample, which is defined as the “CD8+TRG score”. Because of the high clinical correlation between DR and DN, a large number of studies have verified that DN is an independent risk factor for DR and DME, while DR and DME are also independent risk factors for DN. We believe that there is an intersection and relationship between the progress of CD8+T cells in two different organs. This study attempted to validate Hub DECD8+TRGs in three independent datasets of diabetic nephropathy (DN) tissue samples, which are GSE142025 (n=27), GSE30528(n=22) and GSE96804(n=61) respectively. With the help of pROC package, the area under curve (AUC) in the receiver operating characteristic (ROC) curve was calculated to assess the diagnostic value of Hub DECD8+TRGs in GSE30528 and GSE96804. To investigate whether Hub DECD8+TRGs could distinguish early DN from late DN, unsupervised hierarchical clustering analysis was again applied to the validation analysis of the GSE142025 dataset, which was also based on the R-package Pheatmap. The data sets involved in the reference analysis in this study are listed in **Table 1**.

**Table 1:** Datasets implemented for analysis in this study.

Dataset	Platform	Case samples	Control samples
GSE160306	GPL20301	10	9
GSE94019	GPL11154	9	4
GSE60436	GPL6884	6	3
GSE30528	GPL571	9	13
GSE96804	GPL17586	41	20
GSE142025	GPL20301	21	6

### Construction of DR Mouse Model and Verification of Hub DECD8+TRGs Using Quantitative Polymerase Chain Reaction

In addition to comprehensive bioinformatics analysis methods, animal experiments were also executed to verify the reliability and effectiveness of Hub DECD8+TRGs, and the mouse retina was regarded as the research object for qPCR experiment. A total of 29 mice were preferred as experimental subjects, including 17 successfully modeled DR mice, which were assigned to the experimental group, 12 mice were intraperitoneally injected with normal saline during the experiment for “fake modeling”, and they were regarded as the control group. Among them, there were 7 female mice and 10 male mice in the experimental group, 7 female mice and 5 male mice in the control group. The detailed processes of DR mouse modeling is as follows: Eight-week-old C57BL/6J mice (22.5-26g) were fed standard pellet diet without restriction on diet or water. The indoor atmospheric conditions were qualified, the temperature was controlled at (23 ± 2) °C, and the relative humidity was 50%. Blood glucose was checked before modeling (normal blood glucose was between 4.25 and 6.50 mmol/L). STZ solution was prepared by dissolving streptozotocin (STZ, Sigma Company, USA, S0130-1G) in sodium citrate solution of 0.1 mol/L at pH 4.2. Mice were intraperitoneally injected with 55 mg/kg STZ solution for 5 consecutive days, fasted for 6 hours before injection, and blood glucose was measured by tail vein 7 days after the last injection. If blood glucose was > 16.5mmol/L, the diabetic mouse model was considered to be built successfully. Five months after the successful construction of the diabetic mouse model, hyperplasia and disorder of retinal capillaries were examined, and proliferative blood vessel groups were observed in the ganglion cell layer and the inner core layer, indicating that typical characteristic pathological changes of DR had emerged, and the DR mouse model was recognized to be successfully constructed.

TRIzol extraction reagent (TRIzol, Invitgen, Carlsad, CA) was used to extract total RNA from retinal tissue, and reverse transcription was performed with HiFiScript cDNA synthesis kit (First-Strand, Cowin Biosciences, China) to detect the expression level of Hub DECD8+TRGs-mRNA in mouse retina. The primers in the experiment were synthesized by Shanghai Biotechnology Company (Shanghai, China), and the sequence information of the primers is illustrated in **Table 2**. According to the scheme recommended by the manufacturer, the real-time quantitative PCR reaction was accomplished using the PCR system kit SYBGREEN PCR Master Mix (Kangwei Century Co., Ltd., Beijing, China). The internal reference gene opted for measuring the level of gene expression in this study was GAPDH. The relative expression level of Hub DECD8+TRGs was estimated by two power values of ΔCt, and the experiment for each gene in each sample was repeated 3 times.

**Table 2:** Primers for Hub DECD8+TRGs.

Genes	Primer	Sequences
Ikzf1	Forward	CCACAACGAGATGGCAGAAGAC
	Reverse	GGCATGTCTGACAGGCACTTGT
Ptprc	Forward	CTTCAGTGGTCCCATTGTGGTG
	Reverse	TCAGACACCTCTGTCGCCTTAG
Itgb2	Forward	CTTTCCGAGAGCAACATCCAGC
	Reverse	GTTGCTGGAGTCGTCAGACAGT
Itgax	Forward	TGCCAGGATGACCTTAGTGTCG
	Reverse	CAGAGTGACTGTGGTTCCGTAG
Tlr7	Forward	GTGATGCTGTGTGGTTTGTCTGG
	Reverse	CCTTTGTGTGCTCCTGGACCTA
Lyn	Forward	TCCTCAAGAGTGATGAAGGTGGC
	Reverse	ACGGTGGATGTAGTTCTTCCGC
Cd74	Forward	GCTGGATGAAGCAGTGGCTCTT
	Reverse	GATGTGGCTGACTTCTTCCTGG
Spi1	Forward	GAGGTGTCTGATGGAGAAGCTG
	Reverse	ACCCACCAGATGCTGTCCTTCA

## Results

### DECD8+TRGs in the Progress of DME

**Figure 1:**
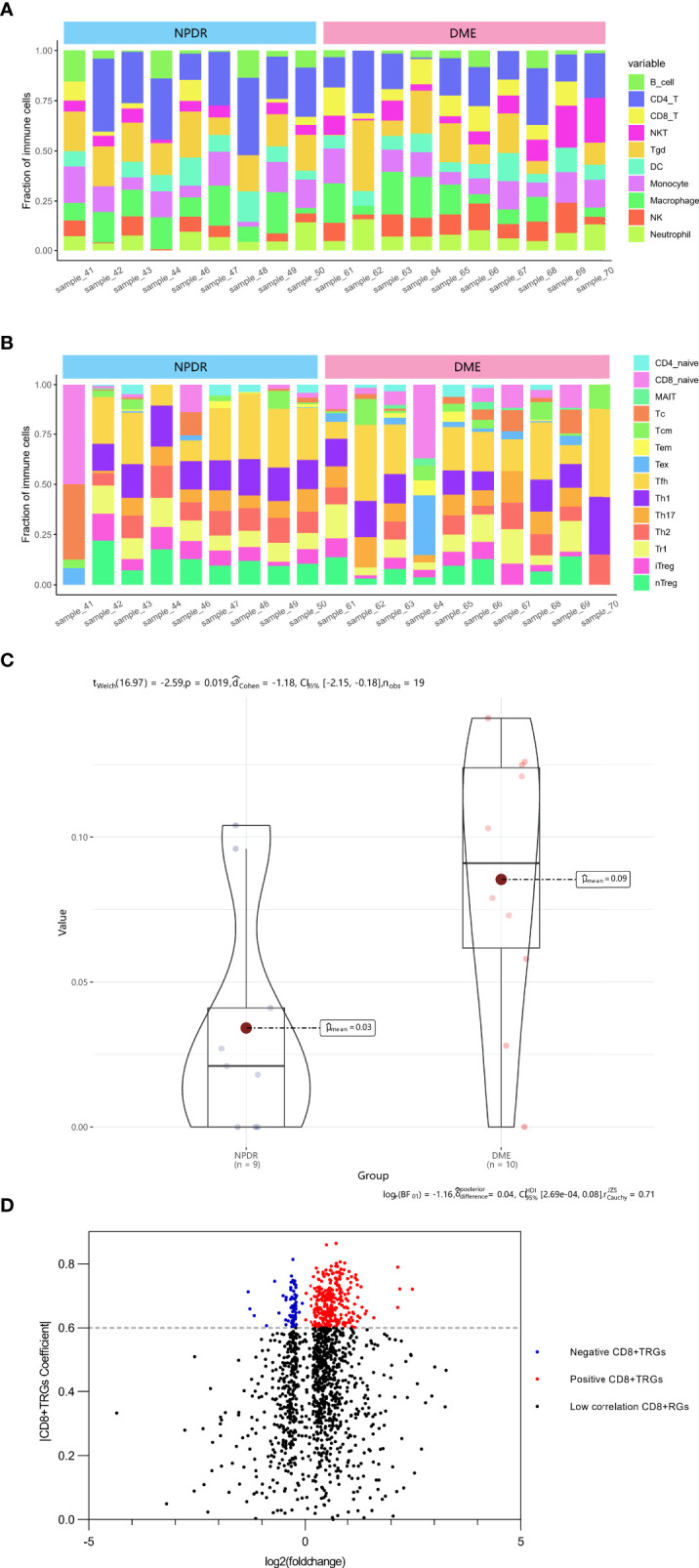
Identification of DECD8+TRGs in macula from DR patients at NPDR stage and DME stage. **(A, B)** The abundance of 24 kinds of immune cells in 19 samples evaluated by ImmucellAI algorithm. **(C)** Overall CD8+T cell proportions comparison between 9 NPDR and 10 DME samples. **(D)** The volcano plot of differentially expressed genes between 9 NPDR and 10 DME samples. DECD8+TRGs, differentially expressed CD8+T cell related genes, CD8+TRGs, CD8+T cell related genes.

The ImmucellAI algorithm has appraised the immune cell composition of each sample in the GSE160306 dataset. **Figures 1A, B** revealed the composition results obtained from 19 macular samples of DR patients. The CD8+T cell infiltration was significantly higher in the DME group than in the NPDR group (**Figure 1C**, p = 0.019). A total of 2221 DEGs were obtained by performing edgeR package for differential analysis combined with correlation analysis (p < 0.05), among which 371 DEGs were highly correlated with CD8+T cell abundance (PCC > 0.6) and were identified as DECD8+TRGs (**Figure 1D**). The abundance of CD8+T cells was significantly different in both NPDR compared with normal and NPDR compared with DME, indicating that CD8+T cells play a pivotal role in the progression of DR and DME. The identified genes were mainly related to CD8+T cells in the progression of DR and DME.

### Enrichment Analysis of DECD8+TRGs

**Figure 2:**
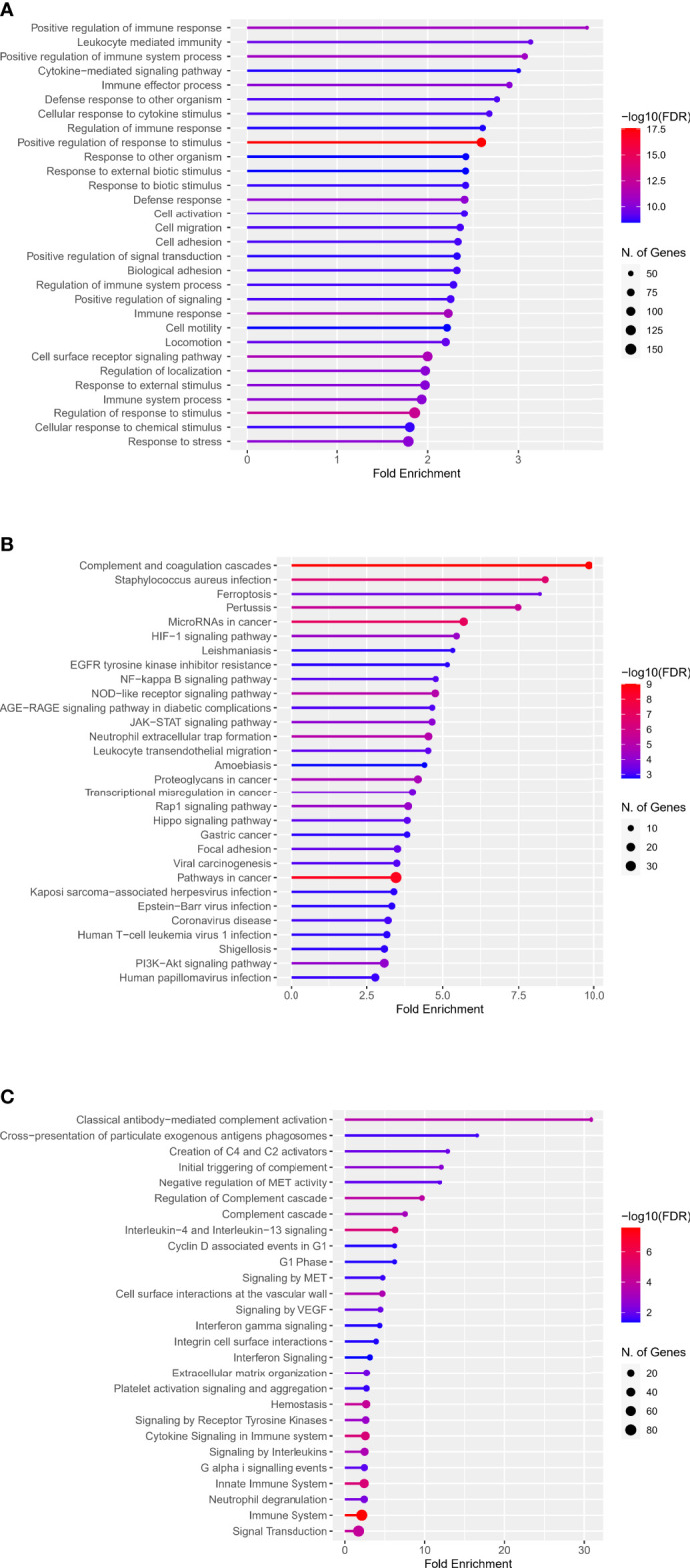
Enrichment analysis of DECD8+TRGs in GO, KEGG and Reactome pathways. **(A)** Top 30 terms of DECD8+TRGs enrichment in the biological process of GO. **(B)** Top 30 terms of DECD8+TRGs enriched in KEGG pathway. **(C)** Top 30 terms of DECD8+TRGs enriched in Reactome pathway. Fold enrichment = GeneRatio/BgRatio.

The web enrichment tool ShinyGO mainly displays the enrichment results from three aspects: GO biological process, KEGG and Reactome pathway. In the biological process of the GO analysis, the top five terms, ranked by Fold Enrichment, were: Positive regulation of immune response, Leukocyte mediated immunity, Positive regulation of immune system process, Cytokine-mediated signaling pathway, Immune effector process (**Figure 2A**). The top five terms in KEGG analysis were: Complement and coagulation cascades, Staphylococcus aureus infection, Ferroptosis, Pertussis, MicroRNAs in cancer (**Figure 2B**). The top five terms in Reactome pathway were: Classical antibody-mediated complement activation, Cross-presentation of particulate exogenous antigens phagosomes, Creation of C4 and C2 activators, Initial triggering of complement, Negative regulation of MET activity (**Figure 2C**).

### Identification of Hub DECD8+TRGs

**Figure 3:**
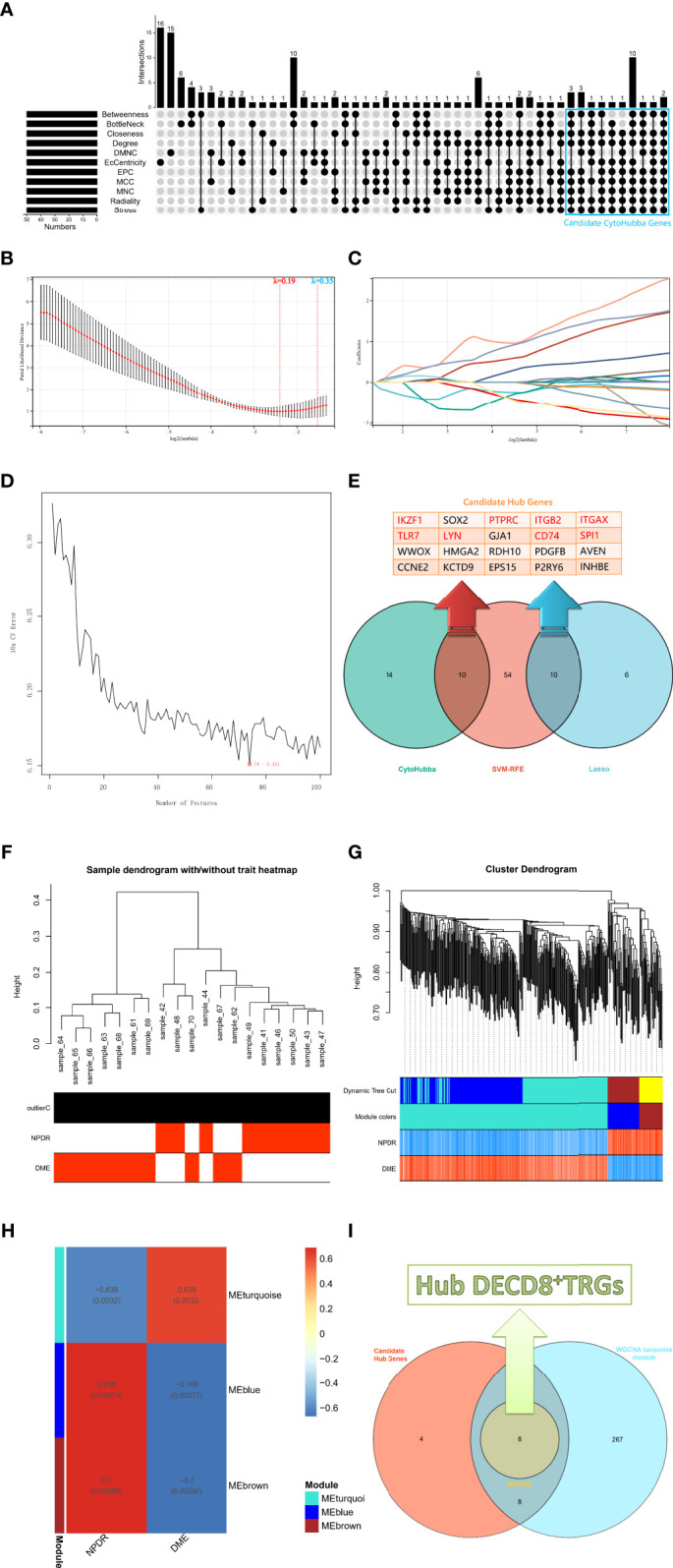
The screening process of Hub DECD8+TRGs. **(A)** The candidate CytoHubba genes obtained by performing eleven algorithms of the CytoHubba plug-in in Cytoscape software, the genes that meet the conditions of 9 or more algorithms were recognized as candidate CytoHubba genes and were marked in the blue box in the figure. **(B)** The parial likelihood deviance in the jackknife rates analysis. **(C)** The Lasso coefficient distribution plot of DECD8+TRGs, which is used to identify the eigenvalues of the constructed diagnostic signal. Each curve corresponds to a candidate gene. **(D)** SVM-RFE algorithm identifies the candidate DECD8+TRGs with the lowest error rate and the highest accuracy (10 × CV error rate = 74 -0.151). **(E)** The Venn plot illustrates the candidate hub genes obtained after the comprehensive intersection of CytoHubba, Lasso regression and SVM-RFE. **(F)** Clustering the samples and checking the outliers. Both NPDR and DME samples were included in the cluster and unsupervised clustering was performed, neither NPDR nor DME sample contained outliers. **(G)** The coexpression gene clusters were analyzed by dynamic tree cutting method, and the modules with high similarity were merged. The hierarchical clustering dendrogram illustrated that there were three co-expression modules, which were MEturquoise, MEblue and MEbrown. **(H)** Clinical feature-related gene module heat map, showing the clinical phenotypic correlation of each co-expressed module gene cluster. **(I)** The Venn diagram shows the genes obtained by the intersection of the candidate hub genes in figure E and the turquoise module genes of WGCNA, in which the core genes are identified by the MCODE plug-in in Cytoscape software and defined as Hub DECD8+TRGs.

The upsetR package in R was executed to visualize the gene intersection obtained by 11 methods in the CytoHubba plug-in, and there were 24 candidate CytoHubba genes that meet the intersection conditions of 9 or more (**Figure 3A**). In the process of building a model by Lasso regression analysis, the candidate DECD8+TRGs that distinguishes NPDR from DME can be accurately estimated when λ = 0.19 (**Figure 3B**). Based on the optimal λ value of 0.19, the LASSO coefficient spectrum of DECD8+TRGs was plotted, and 16 potential candidate DECD8+TRGs were obtained (**Figure 3C**). In the analysis of SVM-RFE, all DECD8+TRGs were included in the operation scope and the SVM-RFE model was constructed according to the top 100 genes of the operation results. The model 10xCV error rate reached a minimum of 0.151 when the number of features was 74 (the location marked by the red circle), at which point 74 genes were recorded and recognized as candidate genes (**Figure 3D**). The genes obtained by the above three methods were summarized after pairwise intersection and were defined as candidate hub genes (**Figure 3E**). All 2221 DEGs were included in WGCNA analysis. The WGCNA package in R was implemented for calculating and plotting. According to the fitting of scale-free network distribution, the correlation coefficient R^2^ was set to 0.9. the optimal soft threshold was automatically set to 18, which ensures high connectivity. No abnormal samples were found when the sample dendrogram was constructed, so no samples were excluded (**Figure 3F**). The correlation matrix between genes and TOM were calculated, and a hierarchical clustering dendrogram was constructed using TOM. The branches of the dendrogram correspond to three different gene modules, each leaf on the dendrogram represents one gene, and similar genes were clustered into modules of the same color (**Figure 3G**). The dynamic cut tree method was administered to identify the gene module, and the modules with high similarity were merged into three modules, meanwhile, the heat map of the correlation between modules and clinical features was also plotted (**Figure 3H**). All three modules were significantly correlated with the clinical features of NPDR and DME (p < 0.001), which were turquoise (p=0.0032), blue (p=0.00073) and brown (p=0.00086). Only turquoise module was positively correlated with the clinical phenotype of DME, while blue module and brown module were negatively correlated with it. Finally, we intersect the candidate hub genes with the three modules in the WGCNA. Only the turquoise module contains 16 intersection genes, while the other two modules have no intersection genes. After that, the MCODE plug-in of Cytoscape software was employed to analyze the intersection genes, and a total of 8 Hub DECD8+TRGs were screened out (**Figure 3I**).

### PPI Network Construction of DECD8+TRGs

**Figure 4:**
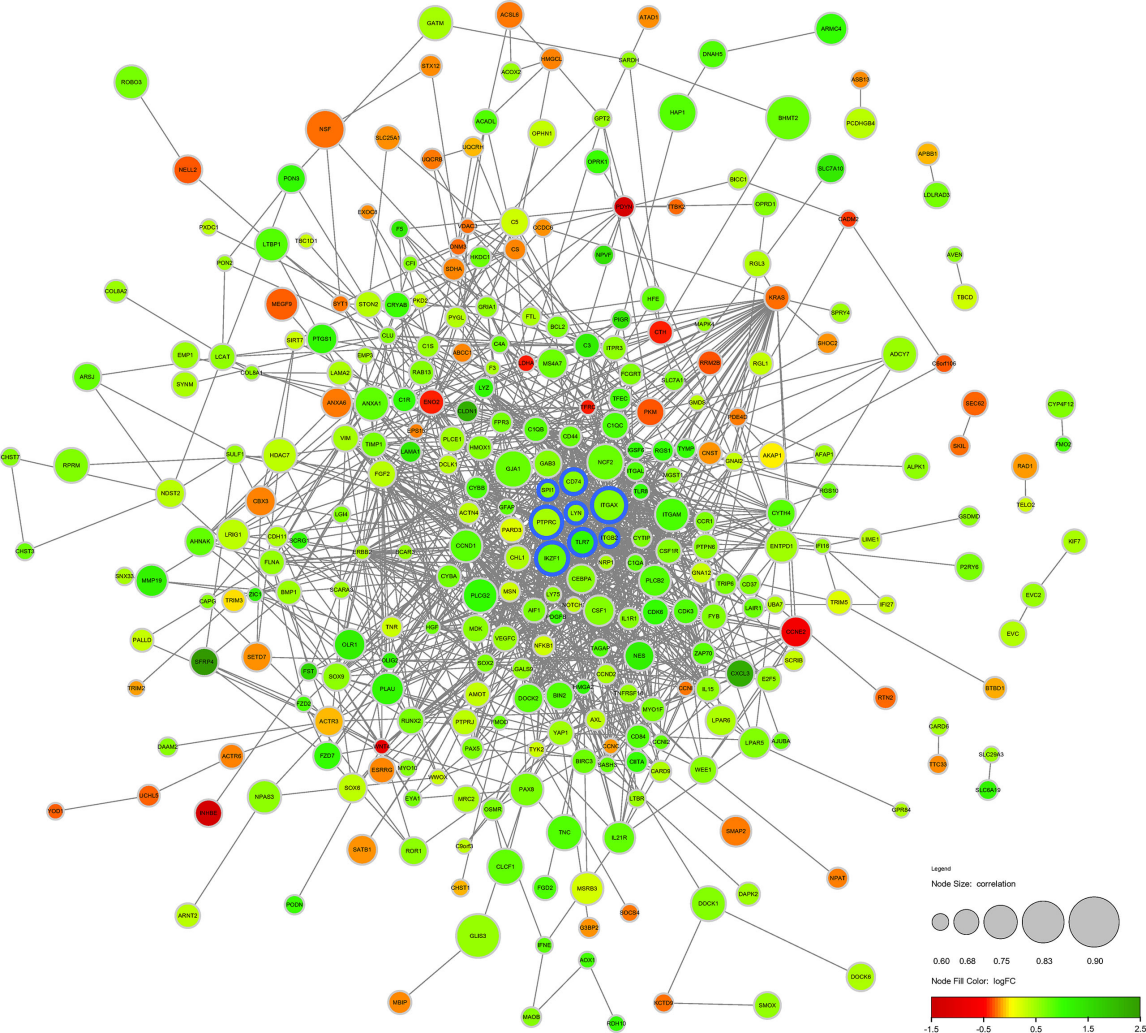
Protein-protein interaction network of DECD8+TRGs. The circular diameter of the node represents the Pearson correlation coefficient between a specific gene and CD8+T cell abundance. The nodes with blue outline represent Hub DECD8+TRGs, the green nodes represent positive logFC, and the red nodes represent negative logFC. FC, fold change.

The DECD8+TRGs protein-protein interaction network, including 371 nodes and 1293 edges, was created by performing STRING online network tool and re-visualized by Cytoscape software (**Figure 4**). After calculation, the degrees of Hub DECD8+TRGs were IKZF1 (degree = 27), PTPRC (degree = 70), ITGB2 (degree = 41), ITGAX (degree = 34), TLR7 (degree = 32), LYN (degree = 29), CD74 (degree = 24) and SPI1 (degree = 38). Compared with NPDR, all 8 Hub DECD8+TRGs in DME were up-regulated.

### Verification of Hub DECD8+TRGs in DR

**Figure 5:**
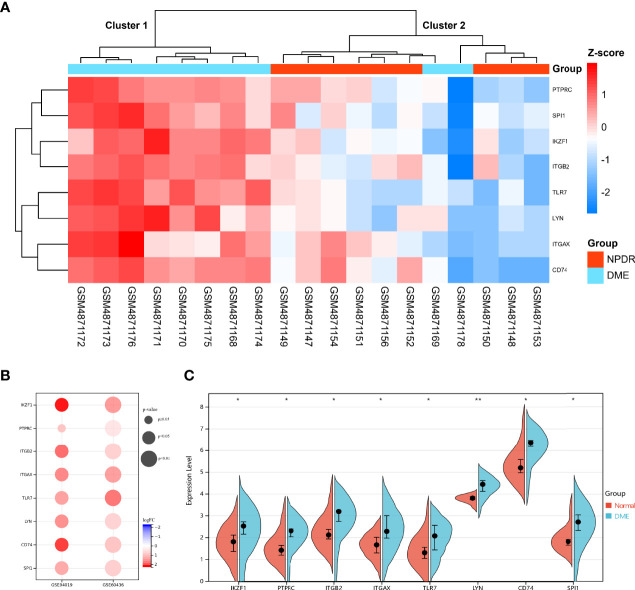
Expression pattern of DECD8+TRGs in DR. **(A)** Unsupervised hierarchical clustering heat map of eight Hub DECD8+TRGs in GSE160306, which illustrates the difference of z-score between NPDR and DME. **(B)** DECD8+TRGs were validated between normal retina and PDR fibrovascular membrane in different independent datasets. **(C)** The difference between normal and DME of eight Hub DECD8+TRGs in GSE160306. *p < 0.05, **p < 0.01.

Hub DECD8+TRGs expression data were extracted from GSE160306 dataset for unsupervised hierarchical clustering. According to the results of ward.D2 clustering method, most DME samples were divided into cluster 1 (8/10, 80%), while all NPDR samples were classified into cluster 2 (9/9, 100%), which fully indicated that these eight genes were not only related to CD8+T cells, but also closely related to the occurrence and development of DME (**Figure 5A**). The expression levels of Hub DECD8+TRGs in the GSE160306 dataset were visualized by grouping violin diagram, which exhibited that there were significant differences in the comparison of eight genes between normal group and DME group (**Figure 5C**). In the process of dataset verification, we perceived that the expression patterns of eight genes of Hub DECD8+TRGs were basically the same in the validation set and significantly upregulated in all validation data sets (except for PTPRC p ≥0.05 in GSE94019 dataset) (**Figure 5B**).

### Verification of Diagnostic Features of Hub DECD8+TRGs in DN

**Figure 6:**
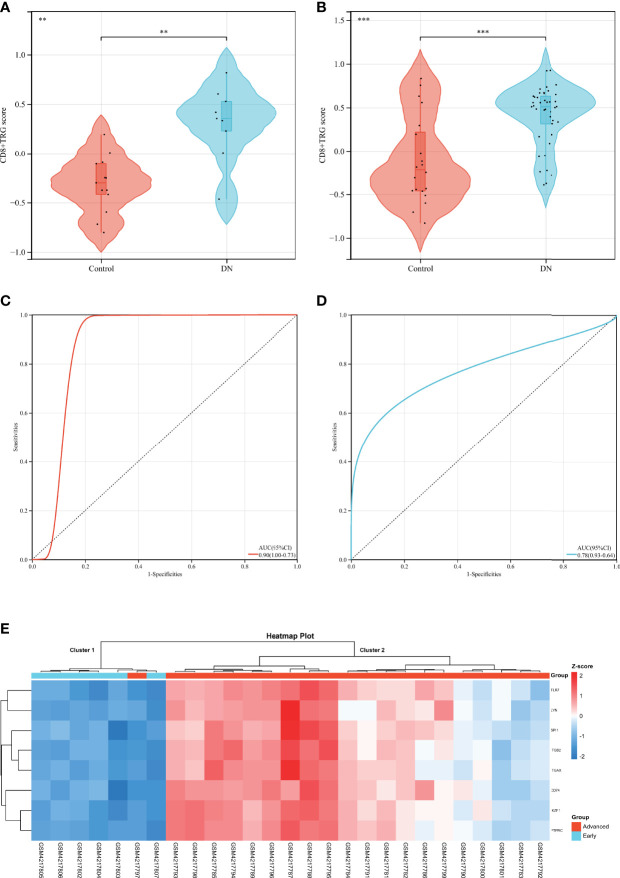
Diagnostic efficacy of CD8+TRG score in patients with DN. **(A, B)** Comparison of CD8+TRG scores between normal and DN in GSE30528 and GSE96804 datasets. **(C, D)** Receiver operating characteristic curve for diagnosis of DN plotted with CD8+TRG score in GSE30528 and GSE96804 datasets. **(E)** Unsupervised hierarchical clustering heat map of Hub DECD8+TRGs, a set of eight genes in GSE142025, showing z-score differences between early and advanced DN. AUC, the area under the curve. ** represents p < 0.01 and *** means p < 0.001.

Complications of diabetes involve multiple organs in the body, CD8+T cells may infiltrate in multiple organs under pathological conditions, and DR is closely related to DN in clinical practice. Based on the above facts, we considered that Hub DECD8+TRGs might serve as a bridge in the pathogenesis of diabetes inflammation, with one end connected with DR and the other end connected with DN. Hub DECD8+TRGs is likely to play a critical role in DN as well. To testify this hypothesis, eight Hub DECD8+TRGs were aggregated and the CD8+TRG score of each independent sample was calculated by the GSVA algorithm, which is used for inter-group comparison. In GSE30528 and GSE96804, CD8+TRG scores in DN patients were significantly higher than those in normal controls (**Figures 6A, B**). ROC analysis illustrated that the AUC of GSE30528 and GSE96804 reached 90% and 78% respectively, which could be considered as having relatively high differentiation (**Figures 6C, D**). In order to verify whether Hub DECD8+TRGs are capable of performing the same discriminant function in the early stage of DN (early DN) and the progressive stage of DN (advanced DN), unsupervised hierarchical clustering of Hub DECD8+TRGs expression data based on GSE142025 dataset was also performed. The results illustrated that all early DN samples were divided into cluster 1 (6/6, 100%), while the vast majority of advanced DN samples were classified as cluster 2 (20/21, 95%). It is fully revealed that Hub DECD8+TRGs have high identification value in the early DN and the advanced DN (**Figure 6E**).

### Validation of Relative Expression of Hub DECD8+TRGs by qPCR

**Figure 7:**
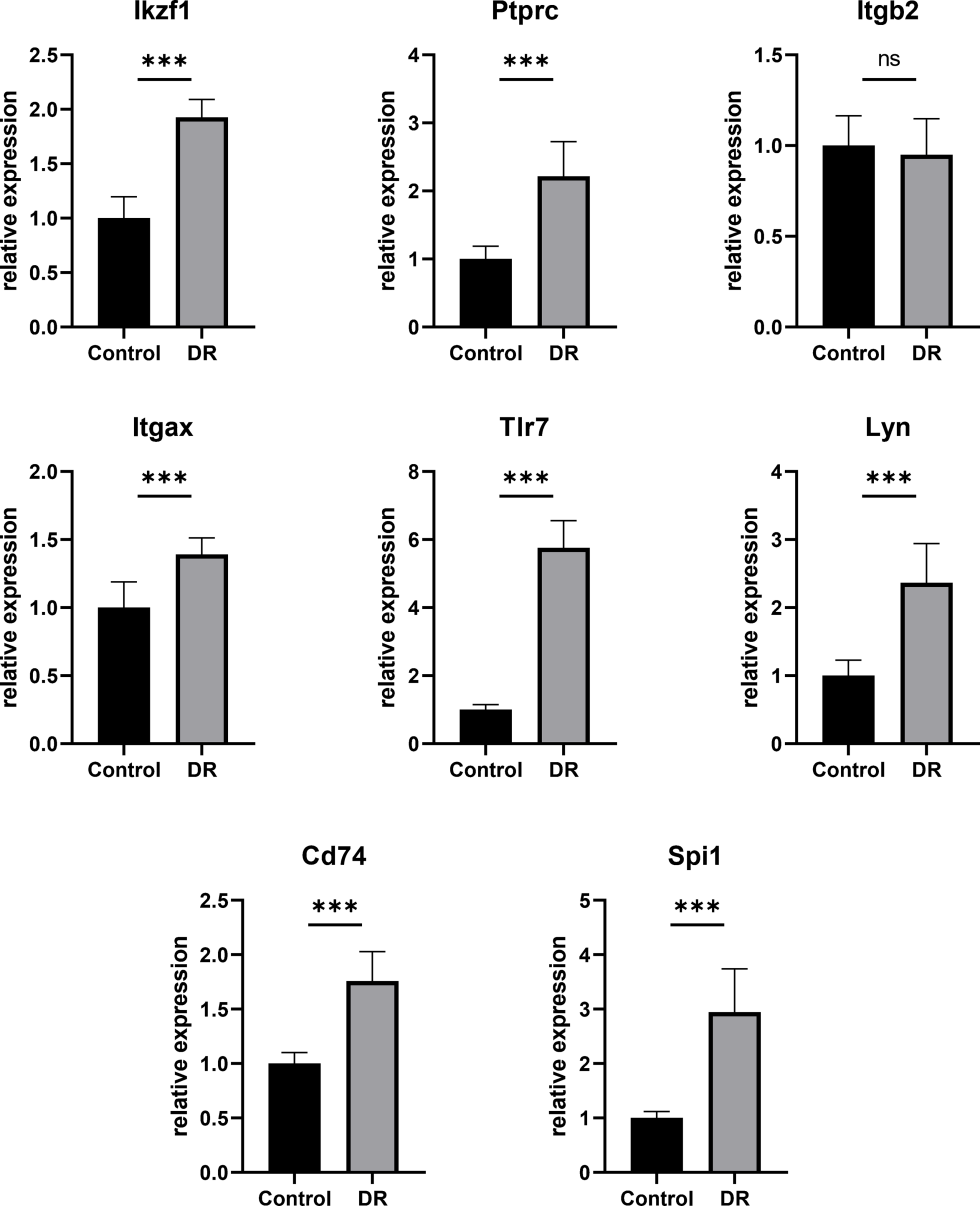
Hub DECD8+TRGs mRNA levels in retinas of DR model mice and control mice validated by qPCR. Compared with the control group, the transcription levels of the other seven Hub DECD8+TRGs in the DR model mouse group except Itgb2 were significantly up-regulated, while there was no significant change in Itgb2. ***p < 0.001, NS p > 0.05.

In order to testify the accuracy of bioinformatics analysis of Hub DECD8+TRGs results, the retina samples were verified by qPCR with the successfully modeled DR mice as the experimental group and the normal mice as the control group. The qPCR results demonstrated that Ikzf1, Ptprc, Itgb2, Itgax, Tlr7, Lyn, Cd74, Spi1 in DR group were up-regulated by 1.926, 2.216, 0.950, 1.391, 5.754, 2.366, 1.757, 2.943 times, respectively, compared with the control group. Except for Itgb2, the mRNA expression of the other seven genes in the retina of DR model mice was significantly increased (p<0.001) (**Figure 7**). The results of qPCR validation matched those of our transcriptome analysis with good consistency and significant difference. It also proved the reliability of transcriptome data in this study.

## Discussion

CD8+T cells, derived from bone marrow hematopoietic stem cells, mature by gene V (D) J recombination and positive/negative selection in the thymus, and circulate between blood and lymphoid organs after moving out of the thymus (28). There are only 10 ~ 1000 resting CD8+T cells that recognize specific antigens, which ensures that a limited number of CD8+T cells *in vivo* have the ability to recognize a multitude of antigens (29). CD8+T cells are activated *via* the recognition of antigenic peptides presented by MHC I molecules *via* TCR on their surface. The activated CD8+T cells will undergo three stages: expansion, contraction and memory formation (30). A large number of antigen-specific CD8+T cells are produced in the expansion stage, which are able to produce up to 10,000 sub-cells after more than 13 divisions, but 90%~95% of them will apoptosis in the contraction stage to prevent autoimmune diseases. The remaining 5% to 10% of the cells will further differentiate, forming memory cells that survive for a long time and can respond quickly to remove the pathogen when they are stimulated again (31). Current studies have confirmed that CD8+T cells are significantly up-regulated in the vitreous of patients with DR, while CD8+T cells are generally not present in the vitreous of normal human eyes (21). Some researchers have also observed that CD8+T cells are up-regulated not only in the vitreous body, but also in the fibrovascular membrane (FVM) of PDR patients (22, 32). They believe that T lymphocytes play an essential role in the pathogenesis and visual prognosis of PDR (22). Another study indicated that CD8+T cells increased significantly in the FVM of 97% of PDR patients, and these FVM samples were all derived from the active stage of DR (32). An earlier study also found that CD8+T lymphocytes accounted for about 66% of FVM in patients with PDR, but the researchers did not distinguish between active and inactive FVM (33). Meanwhile, some studies have suggested that the presence and density of inflammatory cells, including CD8+T cells, may be a marker for the prediction of visual acuity after vitrectomy (34, 35). Vitrectomy is widely operated by fundus surgeons as the first choice for PDR patients with proliferative vitreoretinopathy, vitreous hemorrhage and even retinal detachment, in order to effectively prevent the progression of the disease and improve the prognosis of visual function in DR patients (36). Visual acuity after vitrectomy in patients with PDR depends on a variety of factors, and studies have illustrated that the most important mission is to preserve the structural and functional integrity of retinal neurons and photoreceptors in the macular region (37, 38). The main factors affecting the integrity of macular structure and function in patients with PDR are: preoperative retinal detachment involving macular, the degree and extent of macular edema, macular retinal ischemia and other conditions affecting blood supply, macular neurons and photoreceptor damage (39, 40). All these factors may lead to the upregulation of intraocular inflammatory cells including CD8+T cells. However, during the occurrence and development of DR, especially DME, research on the molecular mechanism and related genetic markers of CD8+T cell infiltration in retina, especially macular retina tissue, has been stagnated due to the fact that living samples are extremely precious and difficult to obtain. The purpose of this study is to screen and reveal CD8+T cell-associated genes by studying sequencing data from precious human retinas using bioinformatics methods. CD8+T cell infiltration score of DME patients was significantly higher than that of NPDR patients, indicating that the number of CD8+T cells in macular area of DME patients was significantly higher than that of NPDR patients. Based on the enrichment analysis and annotation of DECD8+TRGs, we discovered that DECD8+TRGs were mainly enriched in immune system processes and immune responses along with their regulation, response and regulation of various cytokines, biological and chemical stimulus, cell activation, movement, migration and adhesion; complement and coagulation cascade, leukocyte transendothelial migration, neutrophil extracellular trap formation, and some pivotal pathways; Classical antibody-mediated complement initiation, activation, cascade regulation, cell G1 phase and Cyclin D-related events, IL-4 and IL-13 signal transduction, VEGF signal transduction and so on. Only 8 of the 371 DECD8+TRGs screened by the multiple methods were Hub DECD8+TRGs, which are IKZF1, PTPRC, ITGB2, ITGAX, TLR7, LYN, CD74, and SPI1. Interestingly, these genes screened from DR samples can not only identify the disease grade and progression of DR by verification, but also identify the disease classification and advancement of DN by validation with another method. Crucially, they all displayed a high correlation with CD8 + T cells **Supplementary Figure S1**.

Some studies have declared that systemic inflammation caused by diabetes leads to the destruction of immune homeostasis, its essence is to reset the overall physiological state of patients with diabetes *via* the positive regulation of immune response (up-regulation of immune response), pathological growth of retinal neovascularization, up-regulation of epigenetic events and disorder of cell redox regulation system. This altered pathophysiological environment leads to the development of DR (41). CD8+T cell is a subcategory of leukocyte, so it is logical that these genes are involved in mediating immunity. Studies have illustrated that increased concentrations of various inflammatory mediators and pro-inflammatory cytokines in diabetic eyes are able to activate various related signaling pathways, leading to microvascular occlusion and destruction of the blood-retinal barrier, followed by vascular leakage, lack of capillary perfusion, neurodegeneration, and angiogenesis, which are consistent with the cytokine-mediated signaling pathways in our analysis (42). The activation, movement, migration and adhesion of cells provide the basis for the growth of neovascularization and the formation of FVM framework in DR (43). The adhesion of leukocytes in vascular endothelial cells is a favorable environmental factor for the development of inflammation, and KEGG also demonstrates that leukocytes are involved in transendothelial migration, which are important pathways in the formation and diffusion mechanism of DR inflammation (44, 45). In the results of KEGG and Reactome analysis, we observed that the activation and hemostatic mechanism of coagulation cascade appeared in terms, and their combined effect caused retinal capillary occlusion and even caused large blood vessels such as vein embolism, which is the reason why retinal vein embolism and DR often occur together (46). Both databases also revealed that DECD8+TRGs is related to the activation of complement cascade system, while the Reactome pathway introduced more details of complement cascade in DR, such as classical antibody-mediated complement cascade activation, complement C2 and C4 activation, complement initial trigger, complement regulation and so on, which provide a reference for complement-related research in DR, and related researches are also in progress (47, 48). *In vivo* and *in vitro* studies have reported that high glucose conditions lead to increased release of neutrophil extracellular traps (NET) (49). It has also been reported that the level of NET biomarkers is an independent risk factor for DR, and the presence of NET complex in DM patients with PDR was positively correlated with the severity of DR (49, 50). Multiple factors coordination mediate the ability of neutrophils to exert NET, which has recently been found to be the core participant in the pathogenesis of PDR induced by DM (51, 52). The above facts are highly consistent with the formation of extracellular traps of neutrophils analyzed in this study. At present, a large number of studies have pointed out the key role of VEGF pathway in the formation of DR, and now a variety of commercialized targeted drugs have been introduced, which have made great achievements in preventing DR neovascularization (53, 54). Ferroptosis is a hot research topic in recent years. There are few researches related to DR, and it is in the initial stage of related researches (55). Our results uncovered that DR is associated with ferroptosis, which ranks third in KEGG analysis, indicating that some genes in DECD8+TRGs are closely related to ferroptosis. However, the mechanism of how these genes associate CD8+T cells with ferroptosis in retina is still unclear and worth exploring. The formation of advanced glycation end products (AGEs) is involved in the occurrence and development of diabetic retinopathy. Long-term immersion of the retina in hyperglycemia will accelerate the accumulation of AGEs, which are able to bind to AGEs receptor (RAGE). RAGE is widely expressed in all layers of the retina and up-regulated in the retina of patient with diabetes, and leads to cell damage in all layers of the retina *via* a series of molecular mechanisms, inducing inflammatory response and neovascularization, which is known as the AGE-RAGE pathway in diabetic complications in our KEGG analysis. It plays a central role in persistent inflammation, neurodegeneration and retinal microvascular dysfunction that occur during the progression of DR (56). A number of studies have confirmed that signaling pathways, such as HIF-1, Rap1, NF-κB, NOD, PI3K-AKT, are involved in the pathogenesis of DR and DME (57–60). This study revealed the possible signaling pathways related to CD8+T cells promoting the progression of DME, which provides a basis for follow-up research.

Hub DECD8+TRGs is directly or indirectly related to the pathogenesis of DR. The IKZF gene encodes Ikaros, which is one of the transcription factors in the zinc finger group of the Ikaros family and plays a regulatory role in lymphocyte production and immune homeostasis (61). The ratio of CD4 + cells to CD8 + T cells in the blood of mice with IKZF1 (Ikaros-/-) ineffective mutation was up-regulated, and T cells presented a state of mature deficiency (62). IKZF1 is a candidate pathogenic gene for type 1 diabetes, which is related to its susceptibility (63). The PTPRC gene, which encodes type C protein tyrosine phosphatase receptor, also known as CD45, is expressed in all hematopoietic nucleated cells (64). CD45 regulates the interaction between T cells and macrophages *via* its ligand galactose lectin, which causes the elevation of cytotoxic T lymphocyte-associated protein 4 with CD8+T cell tolerance (65). In the early stage of diabetic retinopathy, a few CD45 positive cells were observed in retinal capillaries, and CD45+ stromal cells were also detected in the epiretinal membrane of proliferative diabetic retinopathy (66, 67). ITGB2 gene, which encodes integrin β-2, also known as CD18, is the β chain of integrin, and it is capable of forming different heterodimeric integrins when matched with other α subunits. The surface expression of β 2-integrin is considered to be a marker of leukocyte activation (68). Macrophage cell antigen 1 (CR3) formed by the combination of CD18 and CD11b is expressed in 7% of cytotoxic T cells, which is one of the subsets of CD8+T cells (69). Increased levels of CD18 in neutrophils were present in each stage of DR and were proportional to the severity of the retinopathy (70). Studies have revealed not only the presence of granulocytes in the vitreous humor of patients with DR but also a marked up-regulation in CD18 levels on their surfaces (71). Animal studies demonstrated that CD18 in retina of DR rats exhibited significant up-regulation and increasing trend within 1 week, 4 weeks and 6 weeks after successful modeling (72). ITGAX gene, which encodes the X chain of protein integrin α, also known as CD11c, binds to the β 2 chain encoded by ITGB2 gene to form leukocyte-specific integrin, which is called inactivated-C3B (iC3b) receptor 4 (CR4). CD11c+CD8+ T cell is one of the members of regulatory T cells, which is very important for the maintenance of immune homeostasis. It has dual characteristics: regulatory activity and effector activity. CD11c+CD8+ T cell not only express inhibitory cytokines, but also act as immune effectors to enhance the immunity of tissue cells (73). Retinal microglia in DR have unique CD11c characteristics. CD11c is expressed in activated microglia, and they have the characteristics of proliferation and migration (74). The expression of CD11c was also up-regulated in M1 macrophages of DR (75). Upregulation of the expression of CD11c on monocytes was also detected in circulating blood of diabetic patients (76). TLR7 gene, which encodes protein Toll-like receptor 7, is a member of the toll-like receptor (TLR) family. It plays a significant role in surveillance and recognition of exogenous invasive pathogens and has the ability to accelerate antigen-antibody immune response (77). TLR7 promotes the reactivity of CD8+T cells *in vivo* and is a potential co-stimulator of CD8+T cell activation and functional regulation. It mainly mediates the enhancement of CD8+T cell function by the following mechanisms: α-CD3 mediates the expression of TLR7 in CD8+T cells, which directly promotes the effector function of CD8+T cells; TLR7 relies on multiple signal transduction pathways, leading to metabolic and immune changes in CD8+T cells, in which glycolysis is the key co-stimulator of cell activation and functional enhancement (78). The up-regulation of TLR7 is a risk factor for DR progression, and its absence reduces the release of proinflammatory cytokine, which in turn reduces inflammation-induced retinal damage, the same effect can be achieved with TLR inhibitors in retinal pigment epithelium (79). LYN gene, which encodes protein tyrosine protein kinase Lyn, is mainly expressed in nucleated hematopoietic cells, nerve tissue (including retinal nerve tissue) and liver. As a non-receptor tyrosine kinase, Lyn participates in the regulation of cell growth, differentiation, adhesion, migration, apoptosis and so on (80). Previous studies have manifested that Lyn plays an essential physiological and pathological role in immune response and inflammatory response (81, 82). Lyn in endothelial cells have the ability to enhance endothelial integrity and maintain vascular barrier (83). The expression of Lyn kinase in CD8+ central memory T cells was increased after stimulation *in vitro* (84). Animal experiments revealed that the expression of Lyn was up-regulated in the retina of DR mice at both mRNA and protein levels (85). CD74 gene, which encodes protein HLA II histocompatibility antigen γ chain, is indispensable in the process of antigen presentation, it plays an critical role in the initiation of specific humoral immune response, and is even considered as a novel type of transcriptional regulatory factor (86). In recent years, it has been discovered that CD74 is also involved in MHC class I molecular presentation of endogenous antigens and cellular immune responses (87). CD74 is also the receptor of macrophage migration inhibitory factor (MIF) and has immunomodulatory function in inflammatory and anti-infective responses (88). Antigen presented *via* CD74 is effective in activating CD8+T cells and enhancing their immune response (89). *In vitro* experiments verified that CD74 could regulate the effector function of CD8+T cells, and there was a significant correlation between CD74 and the presence of CD8+T cells (90). Some studies have suggested that CD74 can be regarded as a marker of microglial activation in diabetic retina (91). Soluble CD74 was significantly up-regulated in the vitreous of patients with PDR. The expression of CD74 was detected in endothelial cells, leukocytes and myofibroblasts in the preretinal fibrovascular membrane, and its expression level was positively correlated with the angiogenic activity of the PDR preretinal fibrovascular membrane (92). SPI1 gene, which encodes transcription factor PU.1, is a member of the ETS transcription factor family (E26 transformation-specific family) and plays a prominent role in a variety of tissues development. In recent years, it has been discovered that PU.1 not only plays a role in the determination and differentiation of hematopoietic lineage, but also plays a role in immunity, adipogenesis, tissue fibrosis and nerve development (93, 94). PU.1 regulates the expression of many inflammatory cytokines. In PU-1 deficient mice, CD8+T cell development was delayed and the number of CD8+T cells downregulated (95). PU.1 participates in the prophase proliferation and late differentiation of T cells including CD8+T cells (94). In the retina of DR mice, Syk mediates the activation of microglia by regulating the transcription factor Pu.1, and the inflammation mediated by the activation of microglia in the retina promotes the progression of DR (85).

In terms of diabetic complications, DR and DN have similar pathogenesis and pathological manifestations, especially in the disturbance of diabetic microcirculation. Microcirculation is the most basic structure of the circulatory system and the main place for material exchange between blood and tissue. Blood microcirculation refers to the circulation of blood in microvessels between arterioles and venules. The abnormal structure and function of microcirculation lead to its failure to adapt to the metabolic level of tissues and organs, affecting the substance exchange of tissues and organ function state, which is called microcirculation disorder (96). Microcirculation disorders caused by diabetes-related factors are called diabetic microcirculation disorders, which not only play an predominant role in the occurrence of diabetic vascular complications, but also participate in the emergence and development of diabetes and insulin resistance (97). The exact pathogenesis of diabetic microcirculation disorder has not been fully elucidated. It mainly involves plenty of functional and metabolic abnormalities, such as vascular endothelial damage, reduction of nitric oxide synthesis, activation of polyol pathway, protein nonenzymatic glycation, oxidative stress, activation of protein kinase C, etc., which leads to microcirculation autonomic dyskinesia, increased microvascular blood flow and pressure (98). Persistent hemodynamic abnormalities may lead to damage of microvascular structure, increase of vascular permeability, exudation of capillaries, thickening of basement membrane, stenosis or even occlusion of vascular lumen, microthrombosis, microcirculatory ischemia and hypoxia, and then lead to organ dysfunction. Clinically, diabetic microcirculation disorder is reflected in diabetic nephropathy, diabetic retinopathy and diabetic neuropathy, which are mainly microvascular diseases (99). Among them, the pathological features of diabetic retinopathy are pericyte loss, vascular endothelial cell apoptosis and blood-retinal barrier destruction, neuronal vascular unit destruction, optic nerve abnormality and angiogenesis (100, 101). The main clinical manifestations of diabetic retinopathy are microaneurysms, bleeding spots, hard exudation, cotton spots, venous beads, retinal microvascular abnormalities and macular edema (102). The pathophysiological basis of DME is tissue fluid leakage, focal edema caused by leakage of microhemangioma and diffuse edema caused by leakage of dilated capillary plexus or arterioles. The cause of leakage is the destruction of blood retinal barrier, which leads to the accumulation of plasma protein and the high osmotic pressure of intercellular matrix, resulting in interstitial edema (103). Vascular endothelial growth factor (VEGF) and other factors are involved in this process. The main pathological changes of diabetic nephropathy are thickening of glomerular basement membrane, deposition of extracellular matrix in glomerular mesangial area, and finally glomerulosclerosis with or without tubulointerstitial fibrosis (104). The main clinical manifestations of glomerular involvement are progressive decrease of glomerular filtration rate and increase of urinary albumin excretion. Microalbuminuria is not only the early clinical manifestation of diabetic nephropathy, but also an important basis for the diagnosis of diabetic nephropathy. Renal tubular injury may occur in the early stage of disease and precede glomerular lesions. Diabetic nephropathy is the main cause of renal failure in patients with diabetes. Normal glomerular capillary act as mechanical barriers, charge barriers, and reabsorption pump, allowing useful substances in the blood to be retained, and harmful substances to enter the urine and eventually be excreted (105). When the renal function decreases, the decrease of serum protein concentration, the outflow of tissue fluid, the formation of tissue edema and the increase of vascular osmotic pressure are the common pathogenesis of retinal and renal diseases (106, 107). Hyperglycemia leads to metabolic abnormalities, enhanced oxidative stress, endothelial damage and release of inflammatory mediators, resulting in the destruction of the blood-retinal barrier and the glomerular filtration membrane barrier, manifested as DME occurrence and decreased glomerular filtration rate (108, 109). In this study, the relationship between Hub DECD8+TRGs and CD8+T cells and its relationship with DR has been partially verified. However, most of the studies on Hub DECD8+TRGs in these two aspects are still in their infancy, and their mechanism in DME is still unclear, which is worthy of further study. In the training set, our study discovered that the expression pattern of Hub DECD8+TRGs between NPDR and DME was consistent with that between normal and DME. In the verification set, we noticed that the expression pattern of Hub DECD8+TRGs in normal and PDR is basically the same as that in the training set in other DR-related independent data sets. The results of the comprehensive study indicate that Hub DECD8+TRGs, which is related to the participation of CD8+T cells in immunologic infiltration in DR, is related to both DME occurrence in the macula and disease progression, as also demonstrated by the brief information on each gene above. More interestingly, the expression pattern of Hub DECD8+TRGs in DN-related datasets is strikingly similar to that in DR-related datasets, whether between early DN and late DN, or between normal and DN. Therefore, the significance of this study is to link the relationship between DR, DME and DN from the macroscopic appearance observed in clinic, and connect them at the microscopic genetic level *via* Hub DECD8+TRGs, which provides a valuable reference for the study of inflammatory infiltration mechanism related to diabetic CD8+T cells.

Despite the application of comprehensive bioinformatics analysis methods, our research still has some limitations. First, under the pathophysiological conditions of long-term diabetes, the gene expression patterns of some immune cells infiltrated around different organs and tissues may be adjusted and changed due to a variety of factors. In this study, immune cells located in glomeruli and macular tissues displayed differences in expression profiles under the regulation of pathological tissue microenvironment. Second, some of the immune cells belong to certain cell subsets (in this study, mainly T cell subsets, including CD8+T cell subsets and CD4+T cell subsets), they may have similar transcriptome characteristics or share part of the common characteristic genes, which may lead to potential interference in the calculation of proportional scores, although ImmuCellAI developers have repeatedly stressed the high accuracy of their analysis results. Third, because the experimental design type of the original data set belongs to the case-control study, it is impossible to clarify the sequential relationship between the expression of DECD8+TRGs and the abundance of CD8+T cells, which is also known as causality. Fourth, it is necessary to conduct a prospective cohort study in diabetic patients without complications such as DR, DME and DN to verify the diagnostic and prognostic value of Hub DECD8+TRGs, and to collect detailed clinical characteristic information of patients to adjust and modify the predictive curve. Fifth, further *in vivo* and *in vitro* experiments are needed to verify the research value of Hub DECD8+TRGs and to further explore its mechanism and the intrinsic relationship between genes, the ultimate goal is to elucidate the mechanism of cross talk of CD8+T cell-related pivotal genes in different organs and tissues of diabetes.

## Conclusion

Through bioinformatics analysis with comprehensive methods and *in vivo* experimental verification, this study has provided possible valuable content for the research direction of CD8+T cell-related genes in the staged progression of DME in DR, and also revealed the possible molecular mechanism of CD8+T cells participating in the progression of DME and DR. This study suggests that eight crucial genes act as clues to CD8+T cells to link the occurrence and progress of DR and DME with the initiation and development of DN. These results expand people’s understanding of the mechanisms by which CD8+T cells play a critical role in diabetic complications such as DR, DME and DN, and provide a blueprint for the development of new therapeutic targets for DR and DME.

## Data Availability Statement

The datasets presented in this study can be found in online repositories. The names of the repository/repositories and accession number(s) can be found in the article/**Supplementary Material**.

## Ethics Statement

The animal study was reviewed and approved by Experimental Animal Ethics Committee of the First Affiliated Hospital of Nanchang University.

## Author Contributions

JH performed the data analysis and drafted the manuscript. JH and QZ contributed to the revising of this manuscript. JH conceived and designed the experiments and revised the manuscript. All authors contributed to the article and approved the submitted version.

## Funding

This study was supported by the Key research and development project in Jiangxi Province, No. 20192BBGL70033, Key research and development project in Jiangxi Province, No. 20203BBG73058 and The Central Government Guides Local Science and Technology Development Foundation, No. 20211ZDG02003.

## Conflict of Interest

The authors declare that the research was conducted in the absence of any commercial or financial relationships that could be construed as a potential conflict of interest.

## Publisher’s Note

All claims expressed in this article are solely those of the authors and do not necessarily represent those of their affiliated organizations, or those of the publisher, the editors and the reviewers. Any product that may be evaluated in this article, or claim that may be made by its manufacturer, is not guaranteed or endorsed by the publisher.

## Supplementary Material

Supplementary Figure S1 The supplementary image shows the PPI graph of Hub DECD8+TRGs.
