# Supplementary figures and images for: CD8+T Cell-Related Gene Biomarkers in Macular Edema of Diabetic Retinopathy
Source: Front Endocrinol (Lausanne). 2022 Jul 22;13:907396. doi: 10.3389/fendo.2022.907396 (PMC9355330; doi:10.3389/fendo.2022.907396)

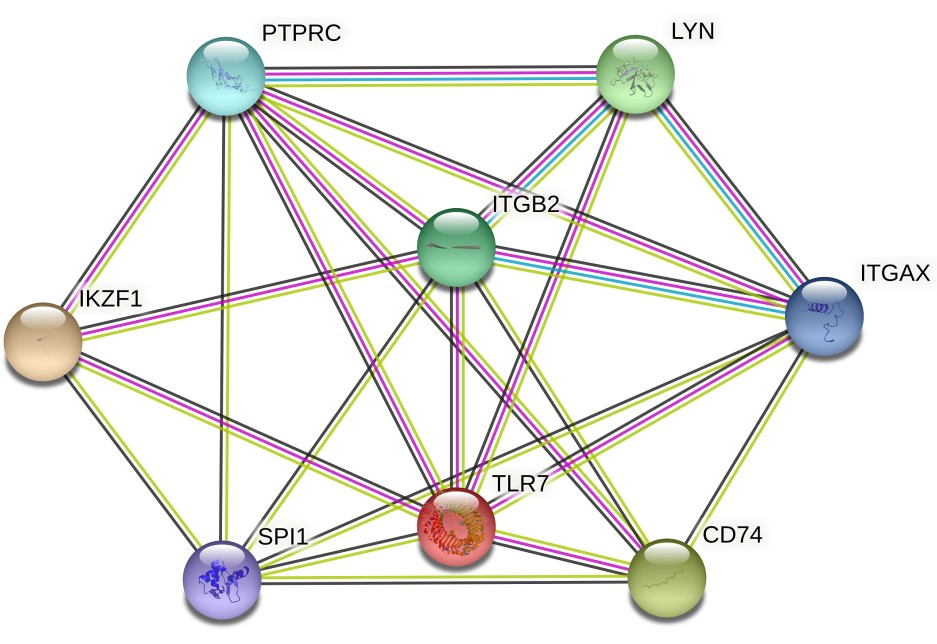

Supplement: Supplementary Figure S1 — The supplementary image shows the PPI graph of Hub DECD8+TRGs. [file Image_1.tif]
